# Sunset Without AIDS: protocol for a randomized controlled trial of a brief video-based intervention to improve the ability of AIDS prevention in elderly men

**DOI:** 10.1186/s13063-022-06069-3

**Published:** 2022-02-14

**Authors:** Jianlan Ren, Mei Li, Yue Luo, Yu Zheng, Jian Tang, Ying Wang, Yanhua Chen

**Affiliations:** 1 Department of Anesthesiology, The Affiliated Hospital of Southwest Medical University, Luzhou, China; 2 School of Nursing, Southwest Medical University, Luzhou, China; 3 Department of Rheumatism and Immunology, The Affiliated Hospital of Southwest Medical University, Luzhou, China; 4 The operating room, The Affiliated Hospital of Southwest Medical University, Luzhou, China; 5 Department of Infectious Diseases, The Affiliated Hospital of Southwest Medical University, Luzhou, China; 6 Department of Nursing, The Affiliated Hospital of Southwest Medical University, Luzhou, China

**Keywords:** Older men, AIDS prevention ability, Video-based intervention, IMB Skills Model

## Abstract

**Background:**

Although progress has been made in the prevention and treatment of HIV in China, there are still a considerable number of new infections annually, especially in older men. HIV health education for older men is urgently needed. Evidence-based, acceptable, and scalable interventions are urgently needed to increase their capacity to prevent HIV. We describe a trial protocol to evaluate the effectiveness of a brief video-based intervention targeting older men's knowledge of HIV prevention.

**Design:**

This study is a randomized controlled trial. The trial will be held in the residents’ activity centers of three communities.

**Methods:**

A total of 450 older men will be randomly divided into three groups (*Sunset Without AIDS* intervention group and two control groups) for 2 weeks. We will assess the feasibility and acceptability of intervention through interviews. The primary outcome includes changes in participants’ knowledge related to AIDS after 2 weeks intervention and 1, 3, and 6 months of follow-up. The secondary outcomes, e.g., changes in participants’ stigma attitude, risk behaviors attitude, and risk behaviors related to AIDS, will be respectively assessed 2 weeks, 1 month, 3 months, and 6 months after the second intervention.

**Discussion:**

*Sunset Without AIDS* may be an innovative way to help older men improve HIV prevention knowledge, fill the gap in video-based HIV prevention education for the older men in China, and gain experience of HIV education. This project will innovate the HIV education ideas of older men and enrich the theoretical research content of AIDS-related education of older men. The findings may also provide the basis for the research and formulation of more reasonable AIDS education strategies, prevention, and control policies for the elderly.

**Trial registration:**

Chinese Clinical Trial Registry ChiCTR2100045708. Registered on 23 April 2021

## Introduction

The AIDS epidemic among older people in China has increased, and the number of new infections has continued to rise. At the beginning of 2017, in China’s 13th Five-Year Action Plan for AIDS Control and Prevention, older people were included in the key education groups for the first time [2]. According to data released by the Chinese Center for Disease Control AIDS, case reports for men over age 60 rose from 8391 in 2012 to 19,815 cases in 2017 [3] and 24465 cases in 2018 [4]. According to the World Health Organization Official WeChat figures released on December 1, 2020, the number of newly diagnosed HIV-infected persons aged 60 and above reported in China in 2019 was approximately 37000, and more than 100 cases were reported daily. This accounted for 25% of the total number of newly reported HIV infected persons. Infected males aged < 60 accounted for 77% of newly reports [5].

The main way of HIV infection in older people in China is sexual transmission, and the imbalance of sexual demand is one of the causes of HIV infection in older people [6]. Studies showed that adults older than 50 years old remain sexually active. Moreover, high-risk sexual behaviors such as having multiple sexual partners and unprotected sex activities with non-mating partners are common in the older population [7]. The survey found that up to 80% of infected older men admitted to having visited prostitutes, while older women were more likely to be infected within marriage [8]. A survey showed that 94.7% of older men infected with HIV never used condoms when having sex with a commercial partner [9]. This shows that older men have a low degree of understanding of safe sex and not notice taking protective measures when having sex with strangers, so they are at risk of infection and transmission of HIV [7]. In addition, for many older men infected with HIV, due to insufficient HIV-related knowledge, traditional sexual concepts, social discrimination, and other existence, they often do not carry out a timely HIV test or treatment and disclosure to their partners or family members. Therefore, it is necessary to educate older men about HIV-related risky sex and improve the AIDS prevention capabilities.

Developing countries represented by China have problems with HIV prevention education, such as outdated education models, poor effect of health education, and uneven distribution of educational resources [10, 11]. Because the literacy level of older people is generally low, it is difficult for them to acquire the correct knowledge of disease prevention. Under the influence of traditional culture, older people generally avoid talking about sex. Therefore, it is also difficult for us to really understand the changes of sexual physiology and psychology of older people. There is an urgent need to develop innovative HIV education model and improve the educational effect, which can be effectively implemented in over-burdened health systems. Video-based interventions are a promising but underused way to address these issues.

As a kind of teaching media, video interventions can deliver standardized messages. At the same time, messages can be woven into engaging storylines, piloted to ensure cultural relevance, and delivered at critical teachable moments [12]. They enrich the integration of education and entertainment. In recent years, some randomized controlled trials conducted in the USA and Africa targeting HIV infected people and high-risk populations. They have found that video-based interventions had a strong track record in improving health knowledge, increasing treatment adherence, promoting HIV screening and reducing sexual partners, supporting partner disclosure, and fostering attitude and behavior change [13–19]. However, there are few evidence-based studies on HIV educational video intervention in China, especially randomized controlled trials to evaluate the effects of video-based intervention to older men.

In recognizing that evidence-based, accessible, and scalable interventions to improve the ability of older men to prevent HIV are critical, our team developed a brief video-based intervention. Below, we describe the protocol of our randomized controlled trial to evaluate its effectiveness to improve older men’s HIV prevention ability in China. This intervention, called *Sunset Without AIDS*, is a 2-week experiment and includes components designed to increase HIV-related knowledge, improve HIV prevention motivation, and strengthen AIDS prevention behaviors among older men.

## *Sunset Without AIDS* overview

### Theoretical framework

In conceptualizing the video, the Information-Motivation- Behavioral Skills Model (IMB Skills Model) was selected. It was based on models which demonstrated the ability to promote behavior change in HIV/STD interventions. This model framework involves three aspects: AIDS-related information, motivation, and behavior skills [20]. *Sunset Without AIDS* includes all components of the IMB model and integrates other evidence-based video techniques to promote behavior change such as video modeling (visual and active demonstrations of desired behaviors) and gain-framed messaging. The combination of *Sunset Without AIDS* and IMB model is shown in Table 1.

**Table 1:** Content of *Sunset Without AIDS* mapped to the IMB concept framework

Component	Story content (18 min)	Expert guidance content (7 min)
Information	The different ways by which the protagonists was infected with HIV and its performance if infected with HIV The older infected with HIV bring many troubles and problems to their families Addressing the misconceptions of AIDS discrimination	Experts provide HIV-related knowledge, attitude, and behavior guidance
Motivation	Avoiding high-risk sexual behaviors will benefit for individuals, partners, and families, thus increasing the motivation to prevent infection The real-life images and characters in the videos (the protagonists are all older) can effectively increase the motivation of older men to watch and learn Positively frames autonomy to facilitate partner disclosure and to seek social support	Analyze the focus issues in the video to strengthen relevant knowledge and behavior training Reiterates key messages using pictures and text to further motivate older men to turn avoiding infection into intrinsic motivation
Behavioral	Protagonist models facing life actively and became AIDS volunteers to contribute their strength to the society	Experts guide on how to avoid infection, such as the correct use of condoms Promotes problem solving to identify ways to reduce discrimination and develop solutions for cross-infection between partners and handle common situations that affect partner disclosure

### Video content development

*Sunset Without AIDS* was developed through an iterative multi-step process involving a team of people living with HIV (PLWH), clinicians, health workers, and other stakeholders. To improve the accuracy of the study, literature research, investigation, and interviews with older people (both male/females), Delphi’s expert consultation method was adopted to determine the educational core content of video design. The development of video content was through a community participatory approach, in-depth research and interviewed with older men and PLWH to ensure that the educational content was acceptable and compelling. Ultimately, the study advisory group identified three key areas that the intervention should target: increase HIV-related knowledge, improve HIV prevention motivation, and reduce high-risk sexual behaviors related to HIV among older men. Intervention video scrip was based on the true stories of old people infected with HIV. The script underwent iterative review and editing by the scriptwriter, until the expert advisory group felt the script addressed these issues in a compelling fashion.

The video was produced through a partnership with Creative Graphic Studio, a company with expertise in making health promotion videos. The result was a film, 25-min long, named *Sunset Without AIDS*. The characters in the video were all experienced actors, and they spoke in Chinese. *Sunset Without AIDS* consists of two parts: one is the story content of approximately 18 min. The other is expert guidance of approximately 7 min. The story content depicted a female protagonist, Li Li, 68 years old, who went to the hospital for examination because she had a cold for several months and did not get better. After being newly diagnosed with HIV, the film portrayed her in a series of complex psychological activities. The cause of her infection was traced to her husband Wang Yong, another 71-year-old protagonist who lost his sense of social existence and value after retirement. At the same time, Li Li often refused to meet her husband’s sexual needs, and the husband believed that he was no longer fertile. Under the egging of his neighbor (a lonely old man living alone), Wang Yong failed to refuse temptation and chose a low-priced prostitute to solve the loneliness. Wang Yong was infected with HIV through heterosexual contact outside marriage and illegal commercial sex without effective protection. Li Li was infected after having marital sex with her husband. At the end of the story, although Li Li and her husband experienced quarreling, complaining, and suffering, they faced the situation bravely with the help and support of their family. They not only received the treatment actively but also acted as volunteers to carry out AIDS health education for the older people around them. Expert introduction regarding HIV-related knowledge, attitude, and behavior guidance was followed at the end of video.

**Fig. 1:**
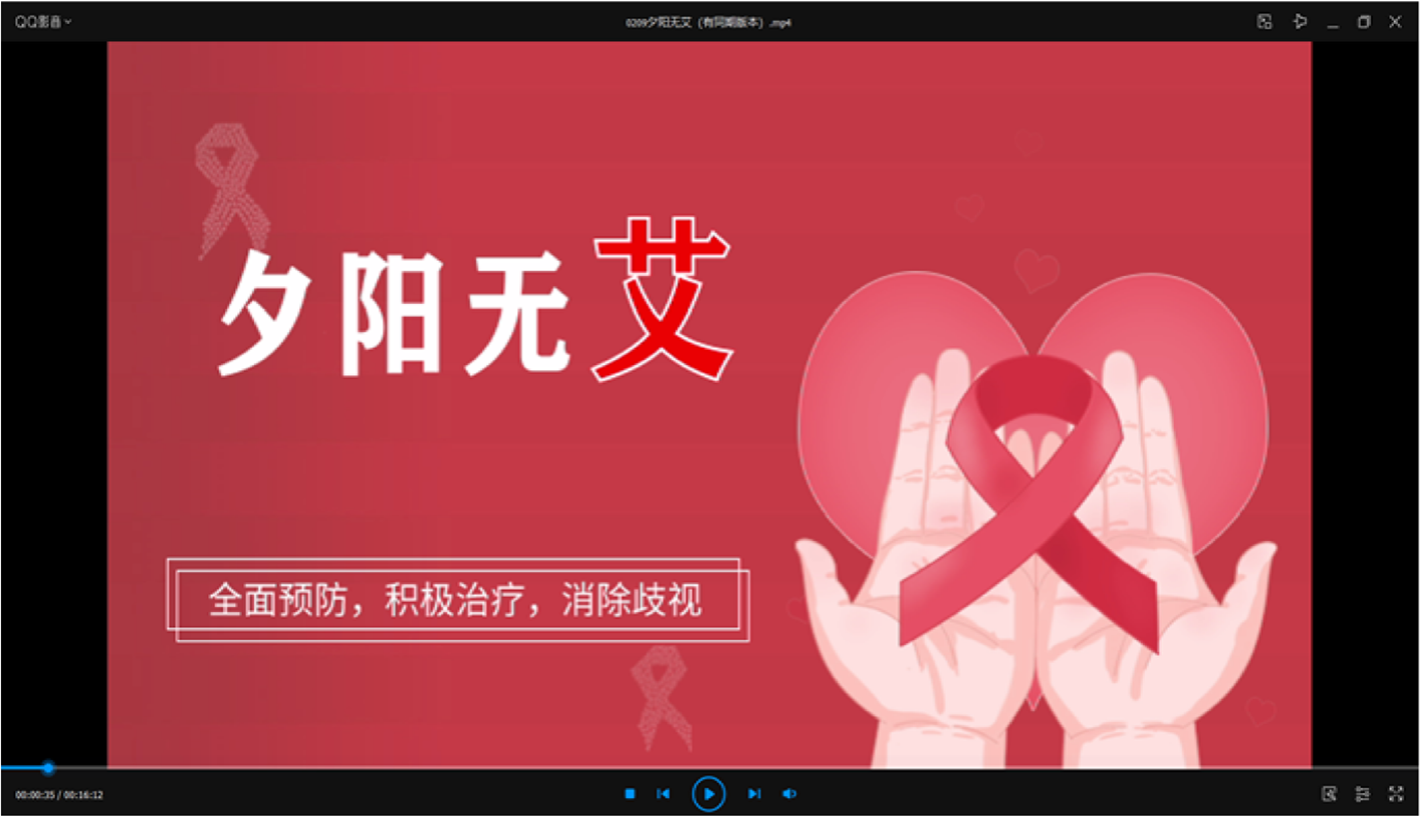
*Sunset Without AIDS*

**Fig. 2:**
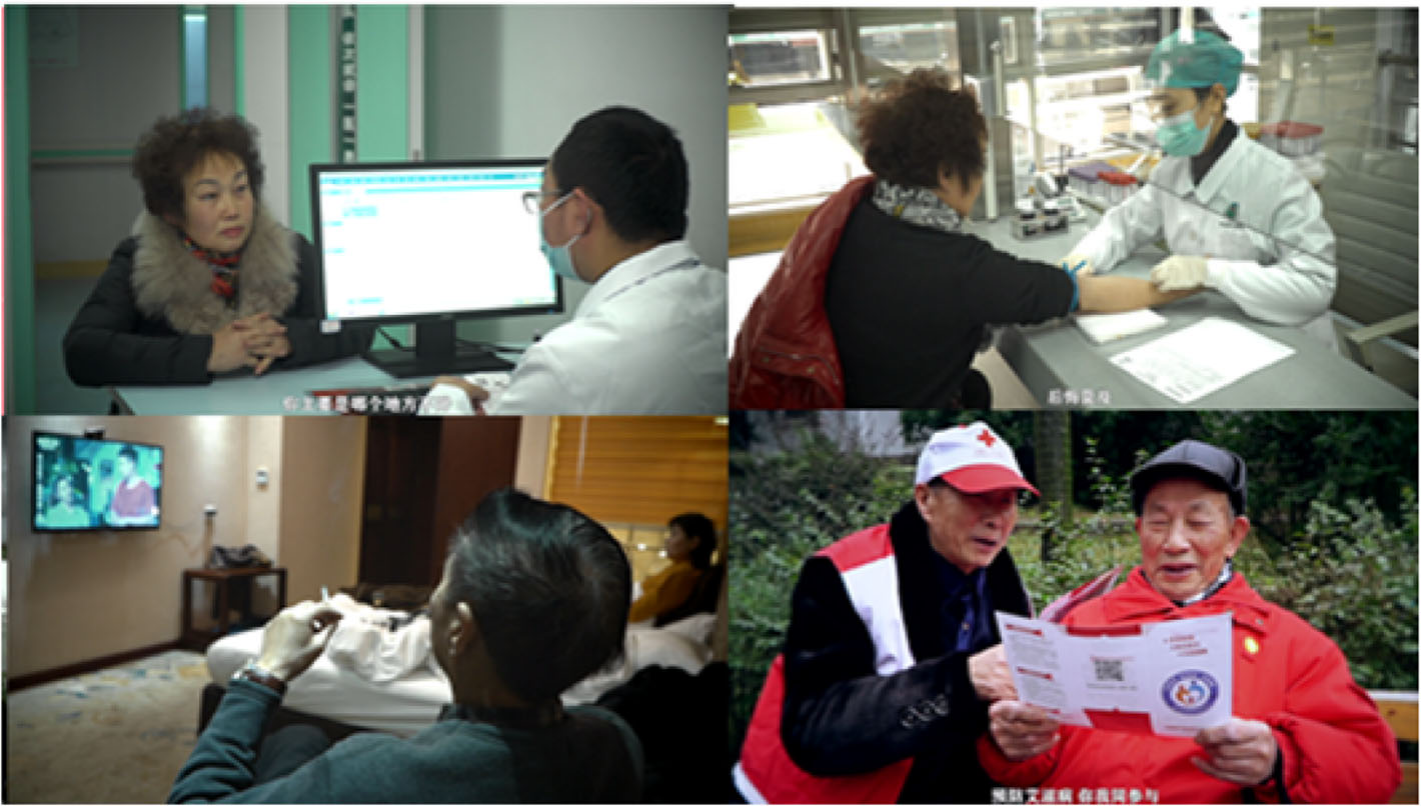
The story content of *Sunset Without AIDS*

**Fig. 3:**
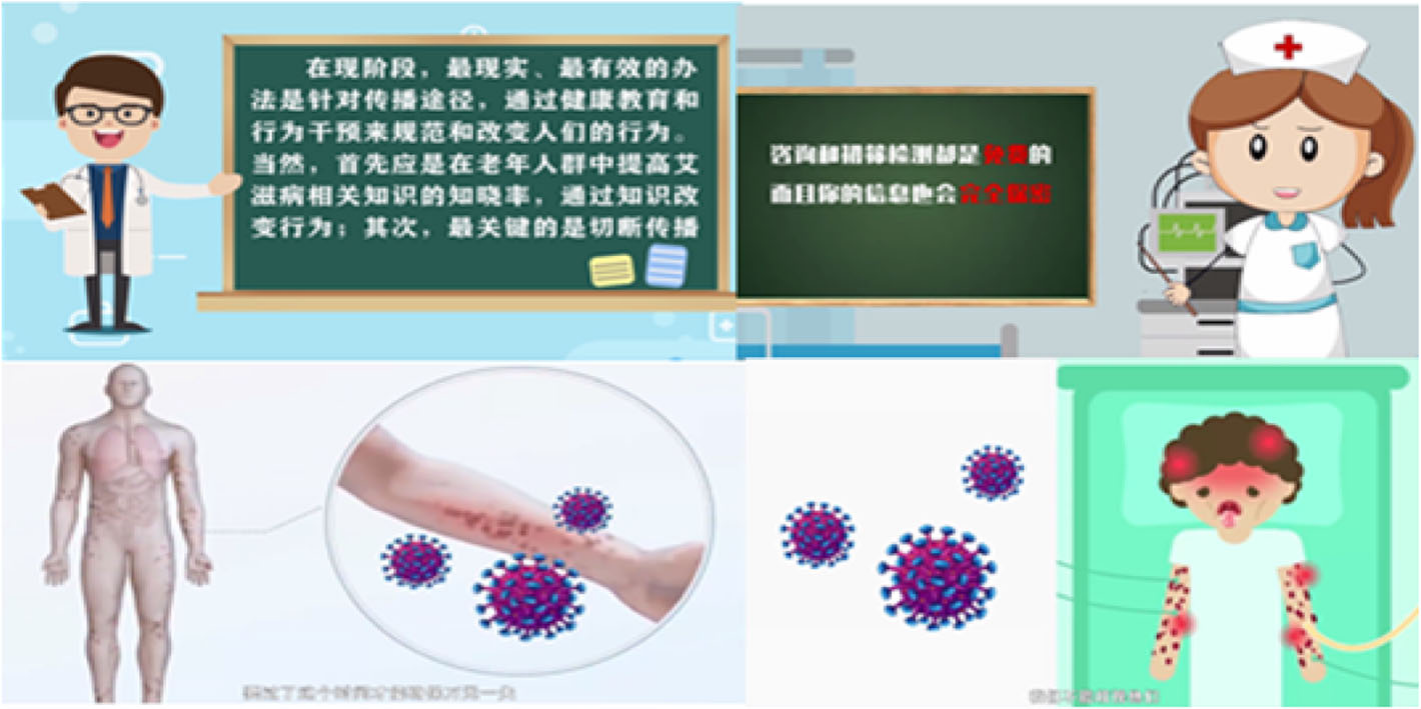
The expert guidance content of *Sunset Without AIDS*

In the *Sunset Without AIDS*, the video provides information about the main route of infection in the older population, the importance of HIV prevention, and the education on HIV testing and treatment. Older men can watch the whole process of HIV infection through this brief video and perceive the risk of AIDS and the severity of the disease, thereby learning knowledge and skills, increasing awareness of AIDS prevention, and reducing risky behaviors (Figs. 1, 2, and 3).

### Aim/objectives

The aim of the study was to evaluate the effect of *Sunset Without AIDS* intervention on improving the ability of HIV prevention in older men. The primary objectives include the following: (1) raising awareness of HIV, (2) reducing HIV-related stigma, and (3) reducing HIV-related risk behaviors.

## Methods/design

Four hundred and fifty participants will be allocated to one of the three randomized groups after completing a baseline survey. Baseline assessments last approximately 30 min and include demographic items, HIV-related knowledge items, HIV-related stigma attitude items, HIV-related behaviors attitude items, and HIV-related behaviors items. At the same time, the acceptability and the method to acquire relating knowledge of HIV prevention will be evaluated through face-to-face interviews with participants.

**Fig. 4:**
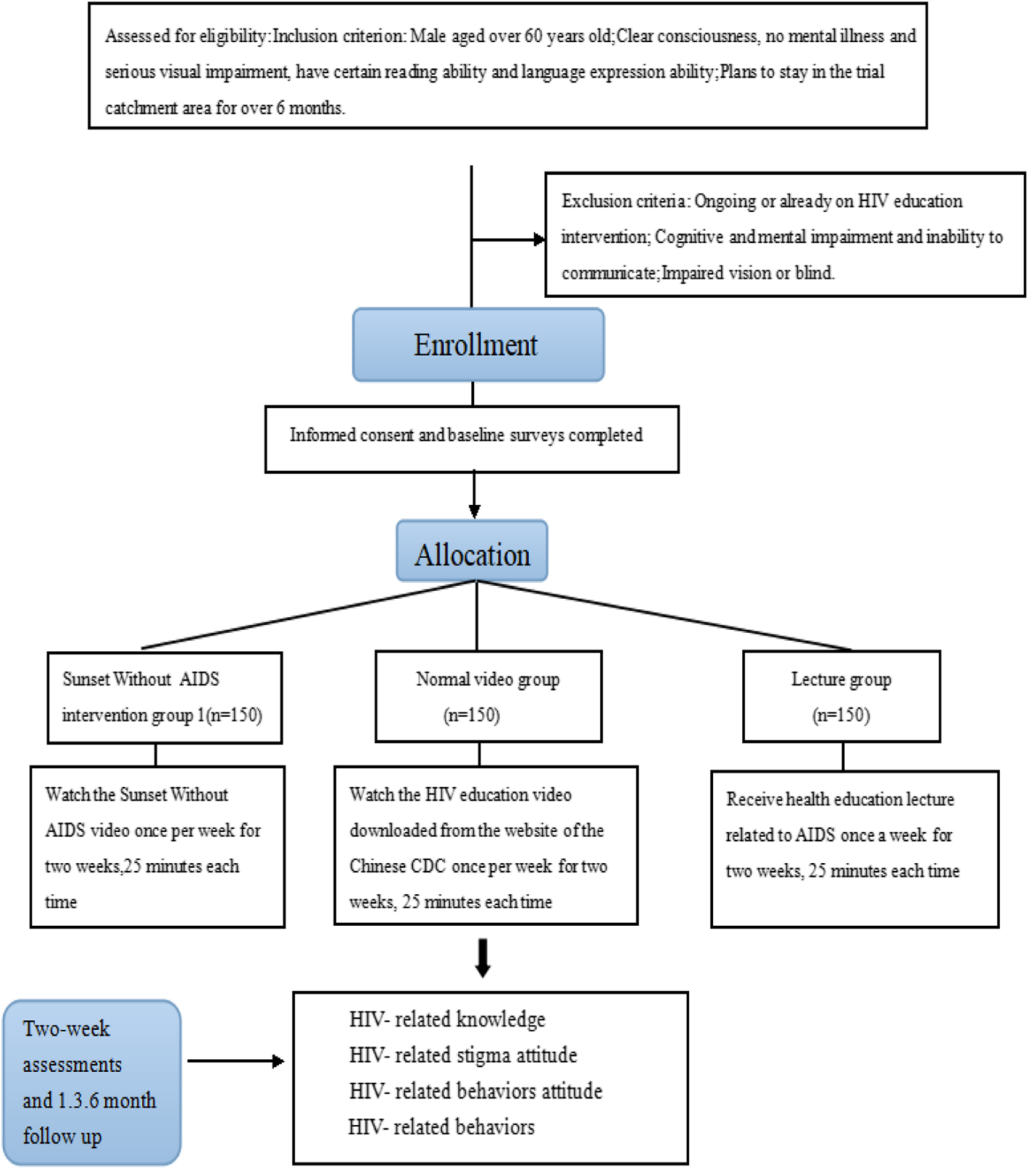
Study flowchart

To reduce contamination, the three groups will be from separate areas. Four follow-up visit assessments are expected at 2 weeks later, 1 month, 3 months, and 6 months. Our hypotheses are as follows: (1) compared with older men in the control groups, those in the *Sunset Without AIDS* intervention group will have a deeper awareness of HIV, less HIV-related stigma, and fewer HIV-related risk behaviors; (2) in comparison between the *Sunset Without AIDS* intervention group and normal video group, the former group integrated various knowledge and behavioral skills into the story. So, it will have better acceptability and feasibility on HIV prevention. Figure 4 shows the study flowchart.

### Trial setting

The trial will take place in Luzhou, a city of Sichuan province in China. Luzhou, with a registered population 5.08 million, located in the southeast of Sichuan Province, is an adjacent city of four province Sichuan, Yunnan, Guizhou, and Chongqing. It is the area where the HIV/AIDS epidemic is rising rapidly in Sichuan Province [21]. Since the first HIV infection was found in Luzhou in 1996, a total of 4688 cases of HIV infection/AIDS were reported from 1996 to 2016, and 49.9% of them were aged ≥ 50 [22].

### Recruitment of participants

We will recruit 450 older men in multiple communities through on-site recruitment. In order to keep the baseline consistent, we have purposefully pre-selected three towns, Fangshan, Jiangbei, and Kuangchang. They are the most representative and have basically the same economic, cultural, and population levels in Luzhou city. In Chinese administrative divisions, each town contains 10–20 communities. In order to facilitate the implementation of intervention, two or three communities will be selected using simple random sampling from each town, and a total of 150 older men will be recruited from these communities in one town. The trial will be held in the residents’ activity centers of three communities (Fangshan community of Fangshan Town, Kuangchang community of Kuangchang town, and Jiangbei community of Jiangbei town).

Participants who sign up will be screened; for those who meet the inclusion criteria, we will issue informed consent to them and inform them in detail of the study purpose and process, number of participants, study duration, potential risks and benefits, random allocation, data confidentiality, etc. All information and written notices will be in Chinese. When the participants agree to join in the research and complete the follow-up measurement, an informed consent form will be signed for confirmation. If the participant is unable to read, the staff will read the informed consent to the participant in full and the participant will mark his or her own fingerprint to express consent; the entire process must be witnessed by a third party not involved in the trial.

To be eligible for trial participation, individuals must meet all the following inclusion criteria:

1. Male aged over 60 years old
2. Clear consciousness, no mental illness and serious visual impairment, have certain reading ability and language expression ability
3. Plans to stay in the trial catchment area for over 6 months

Exclusion criteria include any of the following:

1. Ongoing or already on HIV education intervention
2. Cognitive and mental impairment and inability to communicate properly
3. Impaired vision or blind

The drop out criteria includes any of the following:

1. Have a serious illness during the experiment
2. Death
3. Other irresistible factors, for example, infectious disease isolation

For these dropouts, i.e., those not reaching endpoint, will be counted as failures but analyzed intention-to-treat. It will be counted as having the average score on knowledge score, behavior attitude score, and the stigma scale.

### Sample size and adherence

The sample size calculation is based on the primary outcome, i.e., HIV-related knowledge. The sufficient statistical power to detect changes in HIV-related knowledge after the intervention were considered in sample size calculation, and 135 participants were need in each group to maintain 80% statistical power. Considering this sample size was also powered for the secondary outcomes and the attrition rate, the sample size of each group was determined to be 150, and the total sample size was 450.To motivate participants to stay in the trial, we would use intrinsic reward to help 450 participants adhere to the intervention and follow-up. Participants would receive some prizes for each assessment. In addition, after completing the research, each participant would also receive a reward of 50 RMB in cash.

### Randomization and allocation concealment

Individual randomization will be used in allocation. Participants will be randomly assigned to one of the three intervention groups on a 1:1:1 basis. Opaque envelopes will be prepared for randomization. In 450 envelopes, each will contain a note with the words “*Sunset Without AIDS* intervention group” or “Normal video group” or “Lecture group” (150 envelopes per group). A statistician will be responsible for the generation of random sequence numbers and place them on a sequential list that will be maintained by the trial coordinator. Participants (*n* = 450) will be randomly assigned a computer-generated random number from 1 to 450, and they will randomly select an envelope in the numbering order. For each envelope selected by participants, the total number of envelopes will be reduced by one until all 450 are achieved. Participants will be randomly allocated to the different groups with a closed envelope and supervise from a third party. Separate research staff will record the allocation information of the participants. Another staff will assign participants to interventions. These staff members were not involved in the data collection and analysis process. The data analysts are blinded to the allocation. All interviewers are not involved in delivering the intervention, so they will also remain blinded.

## Intervention group overview

### *Sunset Without AIDS* intervention group

Participants in the *Sunset Without AIDS* intervention group will receive a 25-min intervention. They will be required to watch the *Sunset Without AIDS* video once per week for 2 weeks. During the broadcast, a member of research team will be responsible for answering questions. After 2 weeks of intervention, the effect of the intervention will be evaluated using the same questionnaire as applied in the baseline survey.

To ensure the compliance of participants, participants will be recognized and rewarded after completing two video viewing sessions and 2-week follow-up assessments.

### Normal video group

Participants in the normal video group will receive a 25-min of HIV education video intervention downloaded from the website of the Chinese CDC. And it is a popular science propaganda video, which lacks a storyline and is not specifically aimed at older people. Video length time and duration are the same as the *Sunset Without AIDS* group. The video contains the similar educational contents as the *Sunset Without AIDS*.

### Lecture group

Participants will receive health education lecture related to AIDS once a week for 25 min each time. An AIDS expert will hold a health lecture on AIDS prevention, and the educational content of the lecture is the similar as that in the videos. The whole lectures will be carried out once a week for 2 weeks.

### Contamination and intervention fidelity

To help ensure intervention fidelity and reduce contamination, we developed and revised multiple strategies. Some of the key strategies are listed in Table 2.

**Table 2:** Key strategies to ensure intervention fidelity and reduce contamination

- Increased sample size up to 10% - The study group will discuss the types, scope and measures of reducing possible contamination sources through discussion and literature review - Training of trial staff and health workers to emphasize the effects of contamination. How to avoid it, how to use unbiased explanations to solve the problem - Different intervention groups will be delivered in separate areas - Intervention videos are kept by designated personnel and not available through other channels - Information packs with *Sunset Without AIDS* materials are clearly labeled and stored separately from normal video groups’ materials - Level and extent of participants reported contamination are measured at follow-up visits - Participants will be escorted before grouping and between survey locations - Field study team members confirm that trial participants only receive the specified intervention - Participants are randomly assigned to different groups by selecting envelopes - The data analysts are blinded to the allocation - Ensure that the educational content and duration of the video downloaded from the website of the Chinese CDC is the same as that of *Sunset Without AIDS* - Lecture group will be conduct by an AIDS expert for the same amount of time and content as the intervention group - Intervention sessions audio recorded, and 10% reviewed centrally [23] - All trial investigators will remain blinded	

### Assessments

Once participants’ eligibility is determined, they will be scheduled for baseline assessment. All participants will receive a face-to-face questionnaire independently. After 2 weeks of intervention, the participants will be assessed with the same questionnaire while evaluating the method to acquire relating knowledge of HIV prevention and acceptability of intervention. We will also assess participants using the same questionnaire at 1, 3, and 6 months follow-up after the intervention. All the questionnaires will be provider-administered by interviewers who are independent from the trial, i.e., interviewer asks the participant directly and types in answer. A schedule of enrolment, interventions, and assessments are outlined in Table 3.

**Table 3:** Trial outcomes and schedule of assessments

Measures	0	BL	2 weeks	1 month	3 months	6 months
Recruitment	x					
Eligibility screen	x					
Informed consent	x					
Allocation	x					
Intervention	x					
Demographics		x				
Acceptability			x			
HIV-related knowledge		x	x	x	x	x
HIV-related stigma attitude		x	x	x	x	x
HIV-related behaviors attitude		x	x	x	x	x
HIV-related behaviors		x		x	x	x
Participant satisfaction			x			
Trial fidelity and contamination			x		x	x

### Outcomes and data collection

The primary outcome is HIV-related knowledge; the secondary outcomes comprise HIV-related stigma attitude, HIV-related behaviors attitude, and HIV-related behaviors. The assessment will be conducted in 2 weeks and 1, 3, and 6 months after the second intervention. At the same time, the acceptability and the method to acquire relating knowledge of HIV prevention will be also evaluated after 2 weeks.

HIV-related knowledge will be assessed through a questionnaire. The item of the questionnaire is designed according to the part of the contents for older people in the Core Knowledge of AIDS Prevention And Control Publicity And Education [24] issued by Chinese CDC. There are 10 items in total, one point for a correct answer. The higher the score means the more knowledgeable participants have learned. Six experts were invited to verify the content validity of this questionnaire. The results showed that the item-level CVI (I-CVI) and the scale-level CVI (S-CVI) were both 1.000.

HIV-related stigma attitude will be assessed through the Chinese version of Zelaya’s HIV/AIDS Stigma Scale, which was firstly compiled by Zelaya [25]. The scale, which included 24 items in total, was sinicized by Xing Haiyan in 2014 [26]. In the Chinese version of Zelaya AIDS Discrimination Scale, the Cronbach’s *α* coefficient of the scale was 0.91, and the Cronbach’s *α* coefficient of each dimension was 0.79–0.87. It consisted of four dimensions (fear of infection, stigma prejudice, personal discrimination, social discrimination), with six items for each dimension, a total of 24 items. There were 18 negative items and 6 positive items, and the answers were strongly agree, agree, uncertain, oppose, and strongly disagree. Six positive items are recorded as 1, 2, 3, 4, and 5 points in turn, while 18 negative ones are the opposite. The higher the score of discriminatory attitude, the more serious AIDS discrimination.

HIV-related behaviors attitude will be assessed through an eight-item questionnaire, with one point for each correct answer. The higher the score, the lower the risk of participants engaging in AIDS-related behaviors. Similarly, the content validity of the questionnaire was evaluated, the item-level CVI (I-CVI) was 0.830–1.000 and the scale-level CVI (S-CVI) was 0.875. AIDS-related behaviors will be assessed for whether and how often participants have risk behaviors related to AIDS risk. The fewer AIDS-related risk behaviors the participants have, the better their ability to prevent HIV/AIDS will be.

The HIV-related behaviors will be assessed at 2 weeks later and 1, 3, and 6 months of follow-up after the intervention. The assessment included whether they had sought sexual partners commercially or online and whether they had sex with strangers.

### Statistical analyses

All questionnaires will be administered at baseline, and the final analyses will be adjusted for the baseline scores. Descriptive statistics will be used to show the baseline characteristics of each arm. The outcome measure (HIV-related knowledge, HIV-related behaviors attitude, and HIV-related behaviors) is a binary variable, and it will be compared by chi-square tests among the three arms at 2 weeks later and 1, 3, and 6 months of follow-up. To measure the HIV-related stigma attitude outcome, analysis of variance (ANOVA) will be used to find out whether there are differences among the three arms after 2 weeks of intervention. Repeated measures ANOVA will be used to measure the HIV-related stigma attitude score at 1, 3, and 6 months of follow-up. A Bonferroni correction will be used for multiple testing. Intention-to-treat analysis will be used to account for the influence of attrition. *P* ≤ 0.05 will be deemed statistically significant. All analyses will be performed using SPSS software.

### Monitoring

A third party is responsible for supervising, recording, and processing of data. Blinded data monitoring will be conducted using descriptive statistics for quality assessment purposes and data quality control. We will monitor the intervention on a weekly basis. The level and extent of participants reported contamination are measured monthly at follow-up visits. All unanticipated problems and adverse events will be reported to the principal investigator and appropriate regulatory bodies. All potential problems will be reviewed weekly by the trial coordinator and research supervisors. The data analysts will review trial data monthly.

### Ethical considerations

This trial has been approved by the ethics committee of affiliated hospital of university (No: KY2021071) and the Clinical Trials (ChiCTR2100045708). If the investigators elect to make important modifications to the protocol, amendments will be submitted to both the ethics committee and the Clinical Trials.

### Ancillary and post-trial care

We will answer any HIV- related questions that participants may have during the trial and provide relevant knowledge and psychological counseling after the trial.

### Dissemination policy

Results will be published in papers after the study is completed and reported to the local stake-holders, as well as at local and international conferences. *Sunset Without AIDS* will be broadcast on online platforms. The original data will be uploaded to the Chinese Clinical Trial Registration Center, which can be found by searching for the registration number of this study.

## Discussion

At present, some developed countries have designed and developed AIDS educational videos under the guidance of relevant theories and models, and their research contents mainly focus on the problems of high-risk sexual behaviors, multiple sexual partners, early pregnancy, drug abuse, disclosure, HIV detection, and other issues of PLWH and high-risk groups. Although these videos enhanced the pertinence of educational videos on HIV/AIDS, due to regional and cultural differences, they did not conform to the national characteristics of AIDS infection among older people in China. Their promotion and application were limited. In China, the awareness rate of HIV among older people is low. The HIV preventive education for them is crucial.

To meet the need of AIDS education of older men, a brief video *Sunset Without AIDS* was developed. As mentioned above, *Sunset Without AIDS* can provide knowledge and skills to older men to prevent AIDS. Whether older men will be interested in and using it is the key point to this study. If the video does not appeal to older men, it will not be popularized among them, nor have educational significance. Compared with many other interventions, *Sunset Without AIDS* is designed to be closer to the lives of older people, which may increase viewers’ willingness to watch the video and prevent AIDS.

By evaluating a brief video-based, scalable and locally developed and culturally tailored tool for improving HIV prevention in older men, three arms were designed in this trial, including one intervention arm and two control arms. To critically examine the implementation of *Sunset Without AIDS*, we will also examine participant satisfaction, intervention fidelity, contamination, and time-motion data. By comparing the intervention group with the normal video group, it can be found whether the *Sunset Without AIDS* is attractive and is better than a popular science propaganda video in improving (participants’) AIDS prevention ability. By comparing the intervention group and lecture group, we can figure out whether only a brief video-based intervention is different from a traditional health lecture intervention. By comparing each group before and after the intervention, we can see the difference in the effect of video-based interventions and health lecture intervention in AIDS education. This randomized controlled trial will critically evaluate this video-based intervention to improve the ability of AIDS prevention in older men. It will provide an evidence for the choice of AIDS education methods for older men.

## Limitations

As with any research design, the trial also has potential limitations. In the follow-up observation, the time may be too long to lead to participant loss. We attempted to address this concern by giving each person some prizes to motivate the participants to continue to participate in the study. In addition, if necessary, participants can be traced through the information about the participants set aside on the informed consent.

## Trial status

Chinese Clinical Trial Registry, ChiCTR2100045708 (data assigned: 23 April 2021).

Trial enrollment started in June 1, 2021, and recruitment will be completed in December 2021.

## Data Availability

The datasets used and/or analyzed as well as data collection forms and model consent forms of the current study are available from the corresponding author on reasonable request.

## Abbreviations

IMB: Information-Motivation- Behavioral
AIDS: Acquired immune deficiency syndrome
HIV: Human immunodeficiency virus
STD: Sexually transmitted disease
CDC: Centers for Disease Control
CVI: Content validity index
ANOVA: Analysis of variance
